# Editorial: Pediatric diencephalic tumors: a constellation of entities and management modalities

**DOI:** 10.3389/fonc.2023.1346803

**Published:** 2023-12-14

**Authors:** Antonella Cacchione, Andrea Carai, Veronica Biassoni, Angela Mastronuzzi, Sabina Vennarini

**Affiliations:** ^1^ Department of Onco-hematology, Gene and Cell Therapy, Bambino Gesù Children’s Hospital-Istituto di Ricovero e Cura a Carattere Scientifico (IRCCS), Rome, Italy; ^2^ Oncological Neurosurgery Unit, Department of Neuroscience and Neurorehabilitation, Bambino Gesù Children’s Hospital-Istituto di Ricovero e Cura a Carattere Scientifico (IRCCS), Rome, Italy; ^3^ Pediatrics, Fondazione Istituto di Ricovero e Cura a Carattere Scientifico (IRCCS) Istituto Nazionale dei Tumori, Milan, Italy; ^4^ Pediatric Radiotherapy Unit, Fondazione Istituto di Ricovero e Cura a Carattere Scientifico (IRCCS) Istituto Nazionale dei Tumori, Milan, Italy

**Keywords:** diencephalic tumors, function-sparing, protontherapy, pediatric brain tumors, target therapy

This Frontiers Research Topic encompasses a collection of five papers and is focused on pediatric diencephalic neoplasms, exploring their manifold aspects and their multi-modality management approaches.

The diencephalon, situated in the central part of the brain as a deep-seated midline region, comprises crucial structures and many different tumors can originate in this area (**Figure 1**), such as optic pathway/hypothalamic gliomas, craniopharyngiomas, low and high-grade gliomas, germ cell tumors, Langerhans cell histiocytosis, and pituitary adenomas (1). Each of these tumors exhibits significant differences in terms of biological behavior, symptoms (secondary to the anatomical structures involved), treatment methodologies and prognosis.

**Figure 1:**
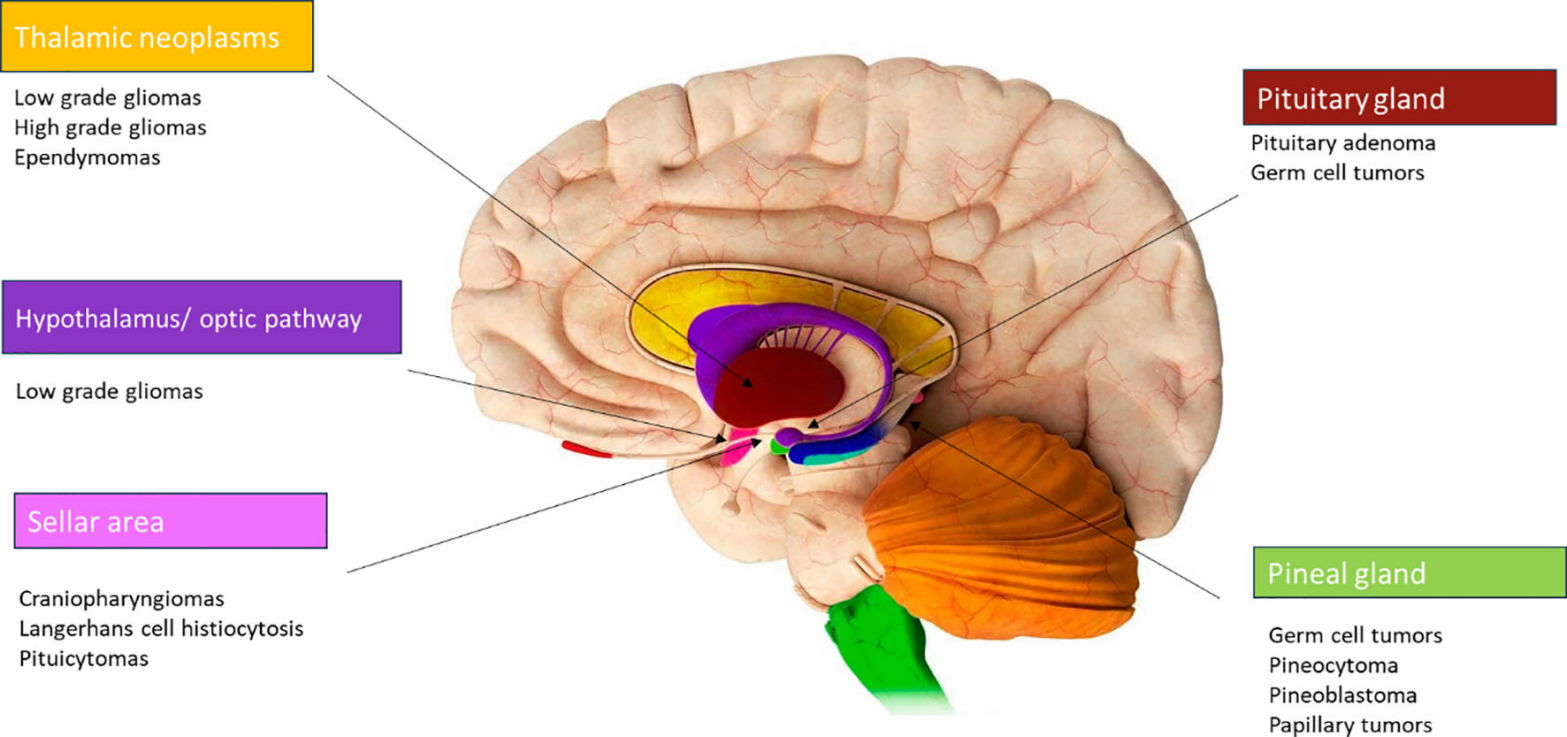
Diencephalon and anatomical areas.

The comprehensive review of Pinto et al. aims to offer a comprehensive overview of diencephalic tumors in the context of the 2021 WHO classification of central nervous system neoplasms (2), providing insights into their epidemiology, clinical presentation, histopathology, along with an exploration of the current strategies employed in their management. These diencephalic tumors can be broadly categorized into four groups:

- Hypothalamic tumors: hypothalamic/optic pathway gliomas are usually low-grade BRAF aberration related. Achieving complete surgical resection of these tumors without significant morbidity is not technically feasible. As a result, chemotherapy remains the primary mode of treatment. Radiotherapy is often avoided due to the significant correlated risks. Patients harbouring BRAF fusions or mutations often respond favorably to therapy with MEK inhibitors or BRAF inhibitors (3).
- Tumors of the neurohypophysis: germ cell tumors, Langerhans cell histiocytosis, pituicytomas and neurocytomas can be found in this zone.
- Thalamic neoplasms: low and high-grade gliomas, ependymomas, embryonal tumors, and neuronal tumors.
- Tumors of the pineal region: germ cell tumors, pineocytomas, pineal parenchymal tumor of intermediate differentiation (PPTID), papillary tumors of pineal gland, and embryonal tumors.

Grippin and McGovern in their paper provide an update regarding the local treatment of these anatomically confined tumors, focusing on the morbidity resulting from the close proximity with critical structures. Due to the outstanding survival outcomes observed in these patients, attention has pivoted towards strategies aimed at mitigating long-term treatment morbidity and the universal agreement that protons (instead of photons) should be used is documented in the Consensus Report from the 2015 Stockholm Pediatric Proton Therapy Conference (4).

In their publication, Lohkamp et al. provide an updated perspective on the multimodal management of craniopharyngiomas (CP), a specific type of diencephalic neoplasm (5). This article aims to recap recent advances in “function-sparing” approaches. Recognizing the importance of tailoring therapies to prevent long-term sequelae, such as endocrine, visual and cognitive impairments, gained prominence after the KRANIOPHARYNGEOM 2000 study. This study emphasized that gross total resection (GTR) does not guarantee a successful cure. In fact, traditionally, the benign nature of CP led to the perception that complete resection was the ultimate cure. However, evolving perspectives over the last decades have shifted towards the awareness that GTR can result in unacceptable hypothalamic injury and that the use of proton therapy (instead of photons) allows to significantly reduce the doses while sparing surrounding normal tissue, resulting in the preferable modality to choose (6).

Del Baldo et al. reported a single-center experience of 17 pediatric patients affected by intracranial germ cell tumors (iGCTs) treated with upfront proton therapy (PT).

Given the high treatment success rates, the primary goal now must be focused on enhancing the quality of life by mitigating the long-term complications (7).

The survival analysis demonstrated an overall excellent prognosis with proton therapy, thus confirming the results of the photon-based phase II COG trial. These findings support the non-inferiority of PT in terms of local control compared to photon-based radiotherapy for similar treatment volumes. They assessed visual, endocrinological, and neuropsychological outcome for all patients. Endocrine dysfunctions and visual morbidities remain commonly ascribed to tumor involvement of the hypothalamic-pituitary axis. Neurocognitive impairment stands out as the most prevalent long-term consequence of radiotherapy, with young age, radiation volume, and higherdoses identified as significant risk factors. The features of PT seem to mitigate the risk of neurocognitive impairment (8).

Cockle et al. in their review have updated the constellation of histopathological entities making up pediatric diencephalic tumors, with a focus on their therapeutic approaches to ensure function preserving management (9, 10).

They highlighted the evolving knowledge of the molecular aberrations underpinning the different type of neoplasms, which offer potential targets for novel therapeutic toxicity-sparing drugs:

- Craniopharyngiomas: apart from the conventional therapies, an overview of recent progress in comprehending the molecular underpinnings of CP is presented. CP typically harbors a CTNNB1 driver mutation, with a subsequent potential use of WNT inhibitors (11). The discovery of elevated levels of IL-6R and IL-6 in both tumor tissue and cyst fluid has sparked interest in tocilizumab (12). The activation of the MAPK/ERK pathway in CP, provides rationale for the utilization of MEK inhibitors (13).
- Low-grade gliomas (LGG): typically exhibit nearly universal upregulation of the RAS-MAPK pathway, providing an opportunity for employing targeted therapies.
- High-grade glioma (HGG): one of the future challenges -also considering their heterogeneity- will be employing a precision medicine approach strategy, allowing patients’ stratification into treatment regimens based on genetic alterations detected within each different type of HGG.
- Germ cell tumors: they present an area with an unmet need for innovative therapies. However, the KIT/RAS signaling pathway has been identified as mutated in over 50% of iGCTs (14).
- Langerhans cell histiocytosis (LCH): the identification of the BRAF V600E mutation in LCH has opened avenues for the utilization of BRAF/MEK inhibitors in the treatment.

Summarizing, diencephalic tumors are intricate midline tumors, and pediatric patients with these tumors typically present with symptoms resulting from mass effect on the hypothalamic–pituitary axis and optic nerve. Surgical resection, within maximal safe limits, is recommended. Molecular analyses allow for the identification of potential targets within specific tumor entities, which may play an important role in disease control. While radiation therapy carries several side effects in the pediatric population, different techniques as proton beam therapy (the main choice for many of these neoplasms) have contributed to higher treatment response rates with reduced morbidity.

## Author contributions

ACac: Conceptualization, Investigation, Writing – original draft. ACar: Writing – review & editing. VB: Writing – review & editing. AM: Writing – review & editing. SV: Writing – review & editing.
